# A Case of Henoch-Schönlein Purpura in a Returning Traveler

**DOI:** 10.7759/cureus.106341

**Published:** 2026-04-02

**Authors:** Vishal Akula, Biruk Amare, Sagah Ahmed, Awol Ali

**Affiliations:** 1 Department of Internal Medicine, Mary Washington Healthcare, Fredericksburg, USA; 2 Department of Infectious Disease, Mary Washington Healthcare, Fredericksburg, USA

**Keywords:** adult iga vasculitis, henoch-schönlein purpura (iga vasculitis), returning traveler, rheum, vasculitic rash

## Abstract

IgA vasculitis, formerly known as Henoch-Schönlein purpura, is an immune complex-mediated small-vessel vasculitis that primarily affects children but may occur in adults, where it carries a higher risk of systemic complications, particularly renal involvement. Adult-onset disease is frequently underrecognized and may lack classic features, instead mimicking infectious, autoimmune, or drug-related conditions, thereby delaying diagnosis. In patients with persistent purpura and an unrevealing initial workup, vasculitis should remain in the differential diagnosis. We present the case of a patient diagnosed with IgA vasculitis following recent international travel, highlighting the diagnostic challenges and clinical reasoning that ultimately led to the diagnosis.

## Introduction

Skin and soft tissue lesions following travel are among the most common concerns encountered in returning travelers [1]. Large dermatologic studies have shown that insect bite reactions, superficial bacterial infections, and nonspecific pruritic eruptions are among the most frequent skin conditions observed in post-travel medical visits [1]. However, any dermatologic presentation accompanied by fever, altered mental status, petechiae or purpura, or other signs of systemic illness warrants prompt evaluation, as these findings may indicate potentially life-threatening conditions [1].

In such cases, it is essential for clinicians to obtain a comprehensive history. This should include details regarding travel location and duration, environmental exposures (e.g., freshwater, seawater, animals, insects, and plants), sanitation conditions (food, water, and sewage), dietary intake (including seafood, undercooked meats, and other high-risk foods), use of drugs or supplements (e.g., kratom or mushrooms), and sexual history. Additionally, standard clinical information, including past medical history, current medications, allergies, and vaccination status, should be reviewed [1].

A thorough physical examination is equally critical, with particular attention to a full-body skin examination. Careful assessment of lesion morphology should be performed, including characterization by type (e.g., macules, papules, nodules, plaques, or ulcers), number, distribution, and location (sun-exposed vs. non-sun-exposed areas). While many dermatologic findings may represent exacerbations of preexisting conditions, clinicians must also consider the possibility of new, travel-associated diseases.

The most commonly reported causes of skin lesions in returning travelers include cutaneous larva migrans (9.8%), insect bites (8.2%), skin abscesses (7.7%), superinfected insect bites (6.8%), allergic rashes (5.5%), rashes of unknown origin (5.5%), superficial fungal infections (4.0%), dengue (3.4%), leishmaniasis (3.3%), dog bites (3.2%), spotted fever group rickettsioses (1.5%), scabies (1.5%), cellulitis (1.5%), and other causes (32.5%) [1].

Overall, the differential diagnosis for post-travel dermatologic conditions is broad, ranging from benign, self-limited processes to severe, life-threatening illnesses that may require isolation, public health reporting, or inpatient management. Some conditions may progress to systemic complications, including organ failure and death, underscoring the importance of timely recognition and appropriate evaluation. In certain cases, hospital admission for further workup may be warranted to exclude serious etiologies.

Here, we present the case of a patient who returned from a one-week trip to Italy and presented to the hospital on the day of her return with a diffuse morbilliform rash. We provide a comprehensive evaluation of dermatologic findings in a returning traveler, ultimately leading to an intriguing diagnosis of an underlying rheumatologic condition.

## Case presentation

A 33-year-old woman with no significant past medical history presented to the emergency department with a rash that initially developed on her lower extremities. She presented on the day of her return from a one-week trip to Italy. She first noticed small, erythematous, slightly raised lesions on her legs during her flight from the United States to Italy. One week prior to travel, she experienced mild rhinorrhea and nasal congestion. Over the course of her trip, the rash progressively worsened, spreading from the lower extremities to involve the upper extremities, trunk, neck, back, as well as the palms and soles. She also reported cramping in her lower extremities, which she attributed to increased physical activity during her trip. Despite the use of topical hydrocortisone, there was no improvement in her symptoms. The patient denied fever, chills, chest pain, shortness of breath, nausea, vomiting, diarrhea, constipation, dysuria, melena, hematochezia, vaginal ulcerations, or discharge. She also denied pain or pruritus associated with the rash. Immunizations were up to date, and she reported no recent sick contacts. She worked as a schoolteacher; denied tobacco, alcohol, or illicit drug use; and had no recent use of prescription or over-the-counter medications aside from topical 1% hydrocortisone cream. She was sexually active with one male partner (her fiancé) without condom use. She denied any personal or family history of autoimmune diseases and reported owning a healthy dog with no tick exposure. She lived in a northeastern U.S. suburb without nearby heavily forested areas and was unaware of any recent tick exposure.

**Figure 1:**
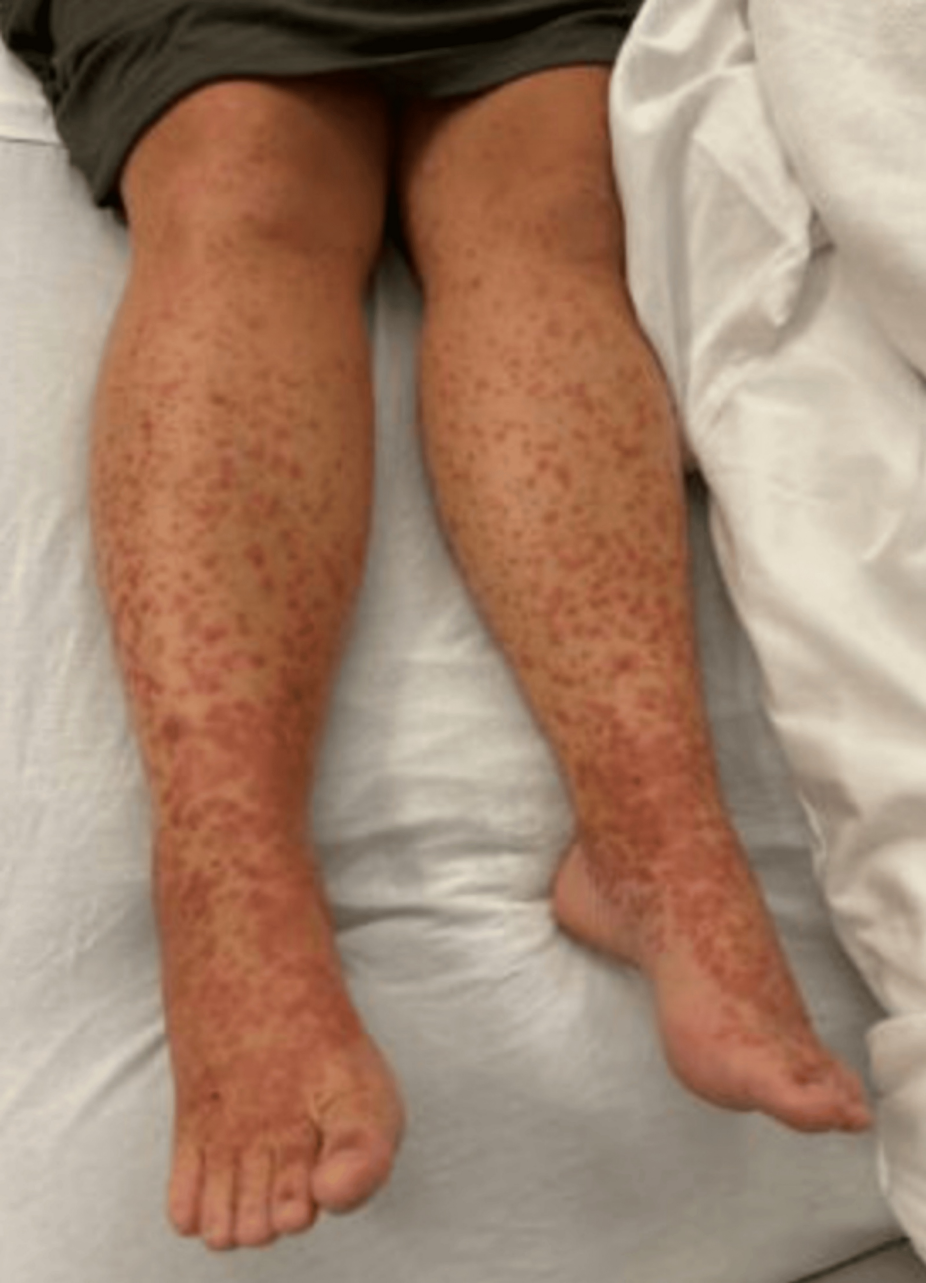
Diffusely morbilliform rash as it demonstrates a diffusely widespread distribution with both macules (flat spots) and papules (raised bumps), which is a classical distribution pattern in IgA vasculitis

**Figure 2:**
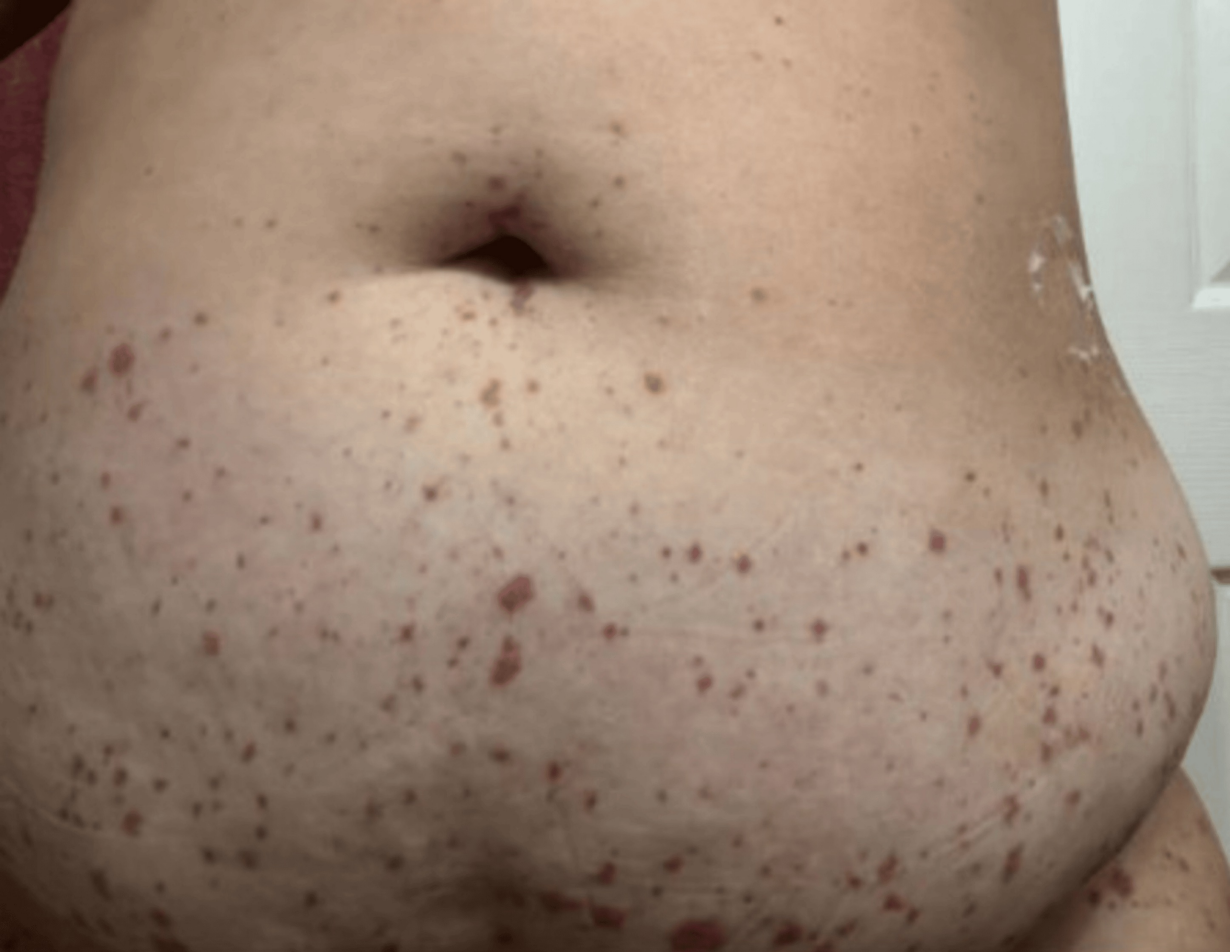
Palpable purpuric (raised bumps) lesions distributed on the trunk, characteristics of IgA vasculitis

**Figure 3:**
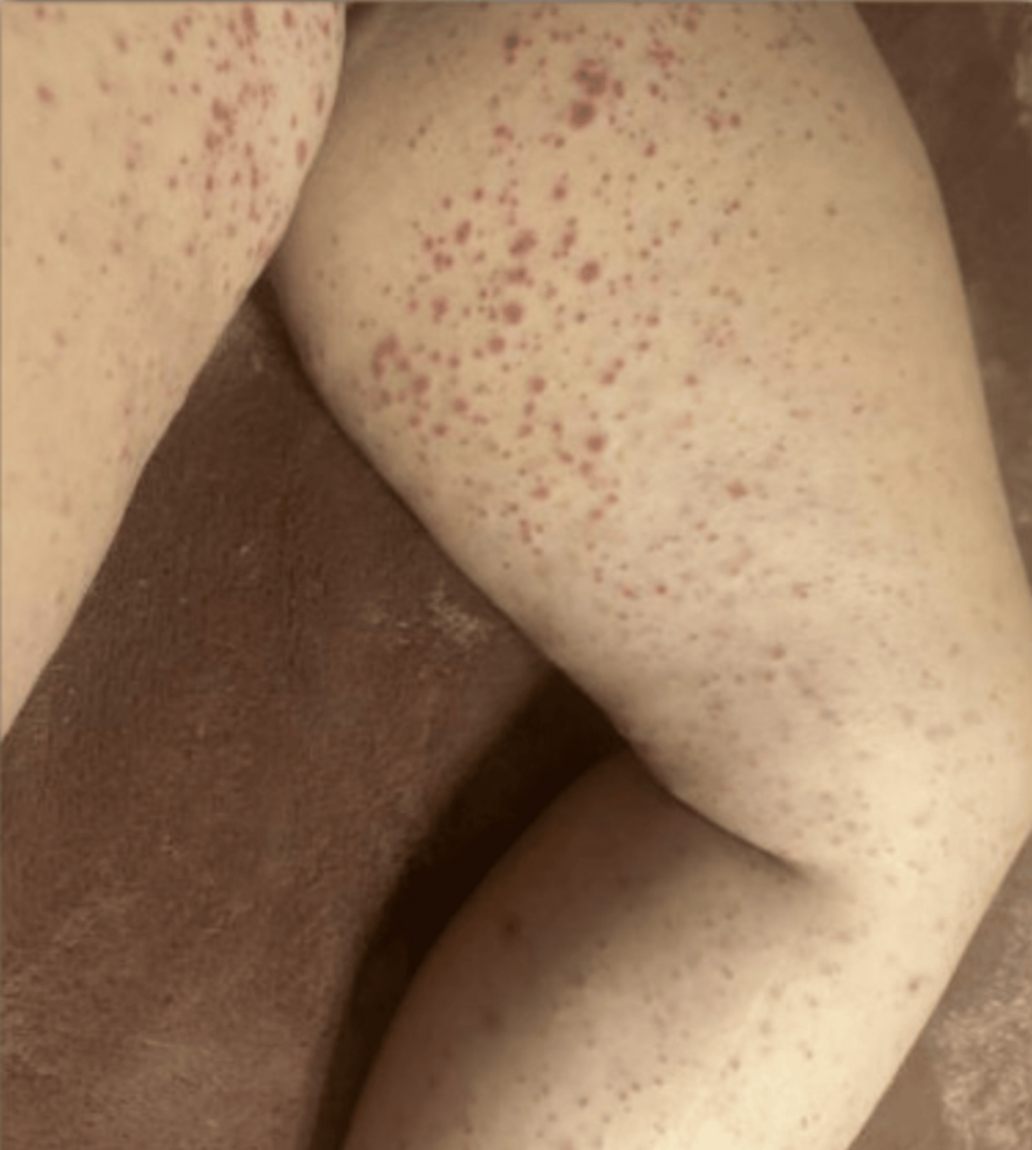
Diffusely morbilliform, palpable purpuric (raised bumps), pink rash on the lower extremity, typical pattern in IgA vasculitis

On presentation, she was afebrile with normal vital signs. Initial laboratory tests, including a complete blood count, a complete metabolic panel, a urinalysis, and a urine toxicology screen, were unremarkable (Tables 1-4). On physical examination, the rash appeared diffusely morbilliform and pink, predominantly on the bilateral lower extremities, extending up the trunk and involving the palms and soles (Figures 1-3).

**Table 1:** Complete blood cell count WBC: white blood cell; RBC: red blood cell; MCV: mean corpuscular volume; MCH: mean corpuscular hemoglobin; MCHC: mean corpuscular hemoglobin concentration; RDW-SD: red blood cell distribution width standard deviation; RDW-CV: red blood cell distribution width coefficient of variation; MPV: mean platelet volume

Parameter	Actual	Normal
WBC	9.31 K/uL	3.4-10.8 K/uL
RBC	4.31 M/uL	3.8-5.0 M/uL
Hemoglobin	12.7 g/dL	11.1-15.9 g/dL
Hematocrit	37%	34.0%-46.6%
MCV	86 fL	79-97 fL
MCH	30 g/dL	26.6-33.0 g/dL
MCHC	34 g/dL	31.3-35.7 g/dL
Platelets	290 K/uL	150-450 K/uL
RDW-SD	40.2 fL	36-46 fL
RDW-CV	12.8%	11.7%-15.4%
MPV	9.3 fL	9.0-12.5 fL

**Table 2:** Complete metabolic panel ^*^Abnormal value BUN: blood urea nitrogen; eGFR: estimated glomerular filtration rate; AST: aspartate transaminase; ALT: alanine aminotransferase

Parameter	Actual	Normal
Glucose	103 mg/dL^*^	70-99 mg/dL
BUN	9 mg/dL	6.0-20.0 mg/dL
Creatine	0.6 mg/dL	0.57-1.0 mg/dL
eGFR	>60 mL/minute/1.73 m²	>60 mL/minute/1.73 m²
Sodium	139 mmol/L	134-144 mmol/L
Potassium	3.7 mmol/L	3.5-5.2 mmol/L
Chloride	105 mmol/L	96-106 mmol/L
Carbon dioxide	23 mmol/L	20-29 mmol/L
Calcium	9.5 mg/dL	8.7-10.2 mg/dL
Anion gap	9 mmol/L	<12 mmol/L
Total protein	7.6 g/dL	6-8.5 g/dL
Albumin	4.1 g/dL	3.8-4.8 g/dL
AST	27 U/L	14-36 U/L
Alkaline phosphatase	59 U/L	38-126 U/L
ALT	28 U/L	0.0-34 U/L
Total bilirubin	0.4 mg/dL	0.2-1.3 mg/dL

**Table 3:** Urinalysis ! represents abnormal values RBC: red blood cell; WBC: white blood cell; UA: urine analysis

Urinalysis	Value
Color, urine	Yellow
Clarity, urine	Cloudy
Specific gravity, urine	1.016
Glucose, urine (mg/dL)	Negative
Ketones, urine (mg/dL)	Trace !
Blood, urine	Negative
Nitrite, urine	Negative
Leukocyte esterase, urine	Small !
pH, urine	5.5
Protein, urine	Negative
Bilirubin, urine	Negative
Urobilinogen, urine (mg/dL)	0.2
RBC, urine	None seen
WBC, UA	6-10 !
Bacteria, urine	Rare
Squamous epithelial, urine	6-10 !
Hyaline casts, urine	None seen

**Table 4:** Urine toxicology screen TCA: tricyclic antidepressant; PCP: phencyclidine

Urine toxicology screen
Amphetamine screen, urine: none detected
Barbiturate screen, urine: none detected
Benzodiazepines screen, urine: none detected
Buprenorphine, urine: none detected
Cannabinoid screen, urine: none detected
Cocaine screen, urine: none detected
Methadone screen, urine: none detected
Methamphetamine screen urine: none detected
Opiate screen, urine: none detected
Oxycodone screen, urine: none detected
PCP screen, urine: none detected
TCA, urine: none detected

Given the acute onset of the rash, involvement of the palms and soles, and recent travel history, infectious etiologies were strongly considered in the differential diagnosis. However, the workup was negative for human immunodeficiency virus, Epstein-Barr virus, syphilis, and hepatitis. The erythrocyte sedimentation rate was within normal limits, while C-reactive protein was mildly elevated (Table 5).

**Table 5:** Inflammatory markers ^*^Abnormal value

Inflammatory markers	Actual	Normal
C-reactive protein	1.4 mm/hour^*^	0.0-1.0 mm/hour
Erythrocyte sedimentation rate	21 mg/L	0-30 mg/L

Due to the widespread distribution of the rash and minimal response to supportive care, suspicion of a vasculitic process increased. Accordingly, vasculitis-specific laboratory testing and a skin biopsy were performed. Both antinuclear antibody (ANA) and antineutrophil cytoplasmic antibody (ANCA) tests were negative (Table 6).

**Table 6:** HIV and autoimmune panel ANA: antinuclear antibody; HIV: human immunodeficiency virus; RNP: ribonucleoprotein

HIV and autoimmune panel	Actual	Normal
HIV 1/2 Ab Ag combo	Negative	-
Myeloperoxidase Ab, S	<1.0	<1.0 No antibody detected
Proteinase 3 Aby	<1.0	<1.0 No antibody detected
ANA	<1:40	Negative
Rheumatoid factor	<10.0 IU/mL	<14.0 IU/mL
RNP Ab	0.3	0.0-0.9
Smith Ab	<0.2	0.0-0.9
Thyroid-stimulated hormone	1.86 uIU/mL	0.45-4.5 uIU/mL

**Figure 4:**
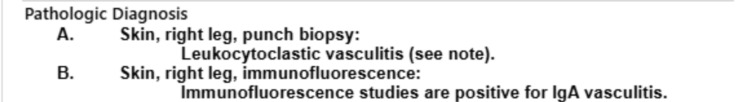
Biopsy results from the lesion on the right leg

Skin biopsy revealed granular IgA deposits with weaker deposits of complement components (C3, C5b-9) and fibrinogen in the superficial papillary dermal blood vessels. Immunofluorescence findings confirmed a diagnosis of IgA vasculitis (Henoch-Schönlein purpura (HSP)) (Figure 4). The patient was treated with supportive care, and the rash resolved over time.

Upon diagnosis, the patient was informed, and a referral to rheumatology was made for close follow-up and further evaluation. Prior to discharge, the rash showed slight improvement, and no additional medication management was prescribed.

## Discussion

This case illustrates a compelling diagnostic pathway leading to the identification of HSP, also known as IgA vasculitis. The patient, a returning traveler presenting with a diffuse, morbilliform rash the day after a trip to Italy, initially raised concern for an infectious etiology, prompting urgent evaluation to exclude potentially life-threatening conditions.

As discussed, returning travelers may present with a wide spectrum of dermatologic conditions, ranging from benign to severe, life-threatening illnesses. Therefore, it is critical for clinicians to prioritize the exclusion of infectious diseases associated with high morbidity and mortality. In this case, the initial workup revealed no evidence of systemic illness or organ involvement, which was reassuring; however, it remained essential to continue investigating the underlying etiology. Clinicians must then broaden the differential diagnosis and consider alternative, noninfectious causes. This approach was appropriately undertaken by the care team. After laboratory studies returned negative and no signs of systemic illness were identified, the next step was to pursue a skin biopsy, which ultimately established the diagnosis of HSP.

HSP remains primarily a clinical diagnosis; however, in atypical presentations, laboratory studies and imaging may provide supportive evidence [2]. An expanded immunologic evaluation, including ANA, ANCAs, rheumatoid factor, and coagulation factors VIII and XIII, may help support the diagnosis or exclude competing pathologies [2]. In this case, all laboratory studies were negative. When diagnostic uncertainty persists or systemic involvement is suspected, imaging studies and tissue biopsy (renal or cutaneous) are indicated [2]. Accordingly, a skin biopsy confirmed IgA vasculitis.

IgA vasculitis is characterized by IgA deposition in small blood vessels, causing inflammation and potential vascular leakage [3]. The classical clinical tetrad includes palpable purpura, gastrointestinal involvement, arthralgias, and renal manifestations [4], with rare pulmonary or central nervous system involvement [3]. Diagnosis typically relies on characteristic purpura, predominantly affecting the lower limbs, plus at least one additional criterion such as abdominal pain, joint symptoms, renal involvement, or histologic evidence of leukocytoclastic vasculitis with IgA deposition [4]. While serum IgA levels may be supportive, they are not diagnostic [4]. In this case, a skin biopsy confirmed IgA deposition, solidifying the diagnosis. Although approximately 90% of cases occur in pediatric patients aged 4-15 years [3], adult presentations are less common and carry a higher risk of renal insufficiency [5].

In retrospect, the patient’s rash demonstrated hallmark features of IgA vasculitis, including its characteristic distribution, coloration, and morbilliform appearance. The occurrence of IgA vasculitis in this adult patient, without renal involvement and in the context of recent travel, highlights the variability and diagnostic complexity of adult-onset disease. Adult patients, particularly women, may have an increased risk of developing hypertension, underscoring the importance of timely initiation of antihypertensive therapy when indicated, along with lifestyle counseling focused on diet and exercise to optimize long-term outcomes [2]. In this case, establishing the diagnosis was especially important, as adult-onset HSP is associated with poorer outcomes. Additionally, the patient’s risk of early-onset hypertension places her at increased risk for long-term comorbidities, including chronic kidney disease, cardiovascular disease, and heart failure. Early recognition and appropriate follow-up are therefore essential to mitigate these risks and improve overall prognosis.

Although the precise etiology remains unknown, IgA vasculitis is thought to be immune-mediated, involving complement activation and neutrophil recruitment. Known triggers include infections, foods, immunizations, insect bites, and medications, with upper respiratory tract infections being the most common precipitant in genetically susceptible individuals [2,6]. In this case, the patient reported mild upper respiratory symptoms one week prior to travel, raising the possibility of a triggering event, though no definitive cause was confirmed.

Management of IgA vasculitis is primarily supportive, focusing on hydration and analgesia, with early involvement of rheumatology and nephrology when renal involvement is present. Therapeutic strategies may include angiotensin-converting enzyme inhibitors, corticosteroids, immunosuppressive agents, or plasmapheresis, depending on disease severity [7,8]. In acute presentations with renal involvement, prompt initiation of corticosteroids or other immunomodulatory therapies may be required.

IgA vasculitis can result in immune-mediated glomerular injury, necessitating routine monitoring with renal function panels and urinalysis to detect proteinuria development or progression. If worsening proteinuria is identified, pharmacologic interventions, such as angiotensin-converting enzyme inhibitors, angiotensin receptor blockers, and/or sodium-glucose cotransporter-2 inhibitors, may be employed to mitigate further renal decline [2]. Adult patients require careful monitoring for potential complications, particularly renal deterioration, persistent proteinuria, and hypertension, with timely pharmacologic intervention and lifestyle counseling as indicated [2].

As a systemic small-vessel vasculitis, IgA vasculitis may also affect organs beyond the skin and kidneys. Gastrointestinal involvement, including macroscopic vasculitis and significant bleeding, can contribute to substantial morbidity [2]. In severe or refractory cases with multisystem involvement, prolonged immunosuppressive therapy may be necessary to achieve disease control and prevent further complications [2].

IgA vasculitis is a well-recognized condition that can range from self-limiting, requiring only monitoring, to life-threatening, with potential organ failure. Given this wide spectrum, timely diagnosis is crucial when the index of suspicion is high, allowing patients to engage in regular follow-up with appropriate specialists to monitor disease progression and optimize quality of life.

In our patient, the initial suspicion was high for an infectious etiology due to her status as a returning traveler, necessitating an extensive workup that fortunately ruled out infection. Despite this, it remained important to pursue a comprehensive evaluation, as atypical presentations can occur even in common diseases. Ultimately, the patient was diagnosed with IgA vasculitis in a benign phase, enabling her to follow up with the appropriate specialists for disease monitoring and management, and to initiate therapy if indicated.

## Conclusions

This case highlights several important clinical principles, including the need for a systematic approach to evaluating rashes in returning travelers and the importance of maintaining a broad differential diagnosis, even when an infectious etiology initially appears most likely. Adult-onset IgA vasculitis, or HSP, may present with classic features such as diffuse palpable purpura, often following an upper respiratory infection-yet diagnostic bias toward travel-related infections can delay recognition of noninfectious causes. The patient’s presentation demonstrates how atypical contexts, such as recent international travel, can complicate clinical assessment, making the careful exclusion of infectious etiologies essential. In this case, a methodical evaluation and multidisciplinary collaboration led to the accurate diagnosis of biopsy-confirmed IgA vasculitis, enabling appropriate management and follow-up. Given that adult-onset disease is associated with poorer outcomes, timely diagnosis is critical to ensure close monitoring, specialist involvement, and implementation of strategies to improve long-term outcomes and quality of life. Ultimately, adopting a structured and comprehensive diagnostic approach enhances clinical accuracy and optimizes patient care in complex presentations.
